# Effect of Retinoic Acid on the Cerebral Vasculature: Analysis of the Vasoactive Response of Smooth Muscle Cells in Normal and Ischemic Contexts

**DOI:** 10.3390/jox15030069

**Published:** 2025-05-10

**Authors:** Manuel R. Pouso, Emanuel Farinha, Henrique E. Costa, Margarida Lorigo, Graça Baltazar, Elisa Cairrao

**Affiliations:** 1 Health Sciences Research Centre (CICS), University of Beira Interior (UBI), 6200-506 Covilha, Portugal; manuel.pouso@ubi.pt (M.R.P.); emanuel.farinha07@gmail.com (E.F.); eloi.costa@ubi.pt (H.E.C.); gbaltazar@fcsaude.ubi.pt (G.B.); 2 RISE-Health, Department of Medical Sciences, Faculty of Health Sciences, University of Beira Interior, 6200-506 Covilha, Portugal; 3 Faculty of Health Sciences (FCS), University of Beira Interior (UBI), 6200-506 Covilha, Portugal; 4 Cloud Computing Competence Centre, University of Beira Interior (UBI), 6200-501 Covilha, Portugal

**Keywords:** cerebral blood flow, neurovascular unit, retinoids, neurovascular diseases, middle cerebral artery

## Abstract

Retinoic acid (RA), a vitamin A derivative, has been shown to prevent the development of neurological disorders by ensuring the integrity of the physiological structure of the neurovascular unit and regulating the physiological cell’s function. After an ischemia event, RA reduces the effects of blood–brain barrier disruption by blocking the apoptotic signaling pathway. However, the effect of RA on smooth muscle cells (SMCs), which are crucial to maintaining blood perfusion, has never been investigated. This study aimed to evaluate the effect of RA on the vasoactive response of middle cerebral artery SMCs in normal and ischemic contexts (O_2_ and glucose deprivation, OGD). For this purpose, SMCs cultures were incubated with RA, and the vasoactive response was evaluated in both conditions (OGD and non-OGD). To simulate OGD, co-cultures of neurons and astrocytes were made and incubated with RA to analyze the effect of the secretome released by these cells on SMCs contractility. In non-OGD conditions, RA induced rapid relaxation of SMCs and, in the long term (24 h), promoted cell contraction. In OGD conditions, SMCs contractility patterns were different when pre-incubated with RA. In these conditions, NA loses its contractility effect, and SNP seems to revert its relaxant effect. However, SMCs pre-incubated with 5 uM RA show the vasorelaxant pattern typical of SNP, despite the OGD condition. These effects demonstrate an effect of RA on the vasoactive profile of SMCs, with therapeutic potential in OGD conditions.

## 1. Introduction

The brain is the most complex organ in the human body and is characterized by its constant energy demand [1]. Therefore, the energy supply is crucial for correct brain activity, since combined with the high energy need, this organ has a limited capacity for energy storage [1,2]. The supply of oxygen and nutrients through blood flow is, therefore, a continuous and extremely organized process, since small changes can culminate in pathological conditions affecting not only the brain but the entire body [1,2]. To satisfy energy requirements, changes in blood flow are controlled by a set of cells that comprise a structure called the neurovascular unit (NVU) [1,3,4]. The NVU is comprised of neurons, glial cells (astrocytes, microglia, and oligodendrocytes) and vascular cells [smooth muscle cells (SMCs), endothelial cells (ECs), and pericytes], playing an important role in maintaining the integrity of the blood–brain barrier (BBB) and the control of cerebral blood flow (CBF) [4,5].

At an arterial level, cerebral blood vessels are composed of three layers. The inner layer includes ECs, which form a physical barrier to isolate the central nervous system (CNS), helping transport substances to the brain and prevent toxins and pathogens in the brain environment [6]. Furthermore, these cells play an important role in the regulation of blood flow through the production of vasoactive factors, such as nitric oxide (NO), which then act on the SMCs [7]. In the media layer, SMCs are mainly responsible for CBF, controlling the blood vessel diameter to ensure that adequate blood pressure is maintained. Moreover, SMCs are also responsible for the distribution of blood flow, thus contributing to the basal vascular tone [8,9]. On the other hand, these cells are also necessary to preserve other functions related to the remodeling of the vasculature after injury. Finally, the most superficial layer, the tunica adventitia, is mainly made up of collagen fibers and fibroblasts [10].

The interruption of blood flow leads to severe neuronal death, since, as mentioned earlier, the brain is highly dependent on a permanent blood supply. This neurological deficit due to inadequate blood perfusion is defined as stroke [11]. Stroke can be described as ischemic (occlusion of the blood vessel) or hemorrhagic (rupture of the blood vessel), with the first type being the most prevalent [11,12]. Ischemic strokes affect roughly 13.7 million people per year and are one of the leading causes of death and disability worldwide [7,11]. During a stroke, several mechanisms are activated, including inflammation, activation of glial cells, increased intracellular calcium levels, and increased permeability of the BBB [11,13]. Treatment options for an ischemic stroke are essentially based on the fibrinolysis of the clot responsible for blood vessel occlusion [7]. However, these treatments have a narrow therapeutic time window, benefiting only a small number of patients, highlighting the need to find safer and more inclusive therapies [7,14]. A possible reason for the delay in developing more inclusive therapeutic strategies may lie in the fact that they focus, essentially, on neuron rescue. Nevertheless, vascular cells are also subject to ischemia and play an important role in the development of brain damage, especially SMCs, which are essential in maintaining adequate blood perfusion [7]. As previously mentioned, the NVU ensures cerebral blood flow control, so the development of therapies must consider the different cellular elements that comprise this unit.

In an ischemic context, astrocytes release several mediators essential for neuron protection and survival [15], as well as harmful factors that compromise BBB integrity and neuron viability [16,17]. Nevertheless, a few minutes after the injury, the injured brain cells produce proinflammatory mediators, cytokines (TNF-α, interleukin (IL)-1, and IL-6), interferon-gamma (IFN-γ) [18], nitric oxide (NO), and reactive oxygen species (ROS) [19]. In addition, it has recently been reported that upon ischemia, factors released by glial cells mediate the vasoactive response of SMCs [13].

Retinoic acid (RA) is the active derivative of vitamin A and regulates gene transcription through the activation of two nuclear receptors, namely the retinoic acid receptor (RAR) and the retinoic X receptor (RXR), playing an important role in neuronal differentiation, as well as in the development and maturation of the vasculature [20,21]. Moreover, RA also appears to modulate the astrocyte-mediated inflammatory response by decreasing the release of proinflammatory cytokines [16,17]. In this sense, several articles have also suggested that this compound is important for optimal functioning in the detoxification system (of xenobiotics or endogenous compounds), where retinoid actions are required to detect, detoxify, and eliminate these compounds. Thus, it has been discussed that RXR (RXRα, RXRβ, and RXRγ) and RAR (RARα, RARβ, and RARγ) can modulate the concentration of some cytochrome P450 (CYP) enzymes, whereby RXR possibly leads to an increase, while RAR leads to a decrease, in the responses of detoxification systems [22,23]. However, it has also been shown that RXR can dimerize with the xenobiotic-sensitive receptors CAR (constitutive androstane receptor) and PXR (pregnane X receptor) and promote an increase in CYP levels related to a decrease in toxic effects on the body [24]. Moreover, RAR seems to be related to the inhibition of these CYP levels, and several xenobiotics have been shown to have RAR agonist activity [22]. In this sense, the study of the effect of RA should always be analyzed from an integrative perspective between physiology, toxicity, and pathology. After an ischemia event, RA reduces the effects of blood–brain barrier disruption by blocking the apoptotic signaling pathway [21]. However, its impact on smooth muscle cells (SMCs), crucial to maintaining blood perfusion, has never been investigated. Although the effects of RA on other NVU cells have been studied, research on SMCs is still needed.

Therefore, to evaluate the effect of RA on the cerebral vasculature, particularly on SMCs, we analyzed how the use of this vitamin A derivative affects the vasoactive response of SMCs from the middle cerebral artery. Furthermore, we also evaluated how this molecule modifies SMC functionality in an ischemic environment by exposing these cells to the secretome of neuron and astrocyte co-cultures, previously supplemented with RA and subjected to hypoxia.

## 2. Materials and Methods

### 2.1. Animals and Ethical Issues

The experiments with animals in this work were performed in accordance with the national ethical requirements for animal research and with the European Convention for the Protection of Vertebrate Animals Used for Experimental and Other Scientific Purposes (Directive 2010/63/EU). All procedures were approved by the UBI Animal Welfare Body (ORBEA, Orgão de Bem-Estar e Ética Animal), with the Approval Code T0023 of 22 March 2022. The rats were housed and acclimatized at CICS-UBI animal facilities, licensed by the Competent National Authority (“Direcção-Geral da Alimentação e Veterinária”, DGAV).

### 2.2. Smooth Muscle Cell Culture

Wistar Han IGS males (n = 10–15) aged 12–16 weeks (Charles-River, Barcelona, Spain) were used to perform the SMC cultures. The anterior, middle, and posterior cerebral arteries are the main arteries that supply the brain. The middle cerebral artery (MCA) is the most frequently occluded by ischemic stroke. Therefore, the culture of smooth muscle cells (SMCs) from the MCA is very important to establish a model to study ischemic stroke. In this sense, cultures of smooth muscle cells were performed according to Cairrao research group [13,25]. Briefly, Wistar males were anesthetized with ketamine (87.5 mg/Kg) and xylazine (12.5 mg/Kg) and then decapitated. The brain was removed and placed in a petri dish with cold phosphate-buffered saline (PBS: 137 mM NaCl, 10 Na_2_HPO, 2.7 mM KCl, and 2 mM KH_2_PO_4_, pH 7.4). Both MCAs were removed using the proper surgical technique and put in a well of a 6-well culture plate (Orange Scientific, Lisboa, Portugal) that had been previously coated with collagen (5 μg/cm^2^). Then, the arteries were placed in an incubator (atmosphere of 95% air and 5% CO_2_ at 37 °C) for 3 min. After this time period, 1 mL of complete culture medium (CCM: DMEM-F12, supplemented with NaHCO_3_ (1.2 μg/L), ascorbic acid (20 mg/L), heat-inactivated fetal bovine serum (FBS; 5%), bovine serum albumin (0.5%), insulin (5 μg/mL), fibroblast growth factor (FGF; 0.5 ng/mL), heparin (2 μg/mL), and a mixture of streptomycin (100 g/mL), penicillin (100 U/mL), and amphotericin B (250 ng/mL), pH 7.4) was added. The explants were placed back in the incubator, and 24 h later, 1 mL of CCM was added again and subsequently renewed every 2 days. Confluent cultures were obtained after 15 to 20 days, and subcultures of these cells were maintained until the fourth passage, all of which were constantly monitored under a microscope. Cell culture is regularly characterized by immunocytochemistry for the SMC marker, α-actin, to ensure the purity of the cultures, as previously described by our group [13,25]. Cultures of SMCs were directly used to assess their vasoactive response in normal contexts by PCSA assays.

### 2.3. Ischemic Stroke Simulation

To simulate an ischemic stroke, primary cortical cultures were performed and further deprived of oxygen and glucose (OGD). These cultures and OGD conditions are described in the following subtopics.

#### 2.3.1. Primary Cortical Cultures

Embryos from 15-day-old Wistar Han IGS rats (Charles-River, Barcelona, Spain) were used to perform the primary cortical cultures (neurons and astrocytes) from the cerebral cortices. Primary cortical cultures (PCCs) were performed by Emanuel Farinha with the support of Genilso Gava-Junior and Graça Baltazar, according to Roque and Baltazar [15]. Briefly, Wistar rat embryos were removed through the abdominal cavity and decapitated. The cortices were dissected and placed in PBS. After mechanical dissociation, the tissue was mechanically separated, centrifuged for three minutes at 400× *g*, and the pellet was then resuspended in Neurobasal Medium (NBM; Gibco, Lisboa, Portugal)—supplemented with B27 (2%; Gibco, Lisboa, Portugal), glutamine (0.5 mM; Sigma-Aldrich, Sintra, Portugal), glutamate (0.5 mM; Sigma-Aldrich, Sintra, Portugal), and gentamicin (120 µg/mL; Sigma-Aldrich, Sintra, Portugal)—and 10% FBS and placed in 24-well culture plates previously coated with 0.1 mg/mL of poly-D-lysine. The cells were maintained in culture in an incubator (95% air and 5% CO_2_, at 37 °C). Subsequently, the culture medium was renewed after 4 days, and the experimental procedures were performed on the sixth day.

#### 2.3.2. OGD and Reperfusion

To simulate an ischemic stroke, primary cortical cultures were deprived of oxygen and glucose. Twenty-four hours before the exposure of neuron and astrocyte co-cultures to oxygen and glucose deprivation (OGD), serum-free NBM was used in place of the culture medium, and 0.1, 1, 5, and 10 μM of RA (Table 1) were added to the cells. These cells were washed twice and incubated with glucose-free Hank’s buffered saline solution (HBSS; 5.36 mM KCl, 4.17 mM NaHCO_3_, 1.26 mM CaCl_2_, 0.49 mM MgCl_2_, 0.44 mM KH_2_PO_4_, 139.9 mM NaCl, and 3.38 mM Na_2_HPO_4_, pH 7.4). After that, the cells were incubated in an airtight hypoxia chamber (Stemcell Technologies) for incubation. The chamber was flushed for four minutes with 20 L/min of a 95% N_2_ and 5% CO_2_ gas combination. After that, it was sealed and kept in an incubator set at 37 °C for four hours. For control conditions, cortical cells were washed twice, incubated with HBSS supplemented with 5.56 mM glucose, and placed in an incubator (95% air and 5% CO_2_, 37 °C) for 4 h [15]. After this period, the medium conditioned by these cultures (PCC secretome) was collected and stored at −80 °C until PCSA assays. To assess the vasoactive response of SMCs in ischemic contexts, contractility studies by PCSA were performed using SMCs pre-exposed 24 h to these secretomes (Table 1).

### 2.4. PCSA Contractility Experiments in SMCs

The cell contractility studies, using planar cell surface area (PCSA), were carried out according to Quelhas, Baltazar [25]. Through the decrease or increase in the cell area, it was possible to evaluate the contraction or relaxation of the cells, respectively. The SMCs from at least three distinct animals were used in this investigation for each condition (*n* = 3 to 4 mice per condition; total *n* = 10), and to ensure the variability of the data obtained, triplicates were performed for each condition tested. After reaching confluence, SMCs were placed in serum-free medium for 24 h to promote the SMC contractile phenotype. The cells were then trypsinized using a solution of trypsin (0.3%) in a Ca^2+^-Mg^2+^- free, phosphate-buffered solution with EDTA (0.025%), plated in Petri dishes previously coated with 5 mg/cm^2^ of collagen and placed in an incubator (95% air and 5% CO_2_, at 37 °C) for 4 h. After this period, the cells were washed 4 times with a Krebs solution (119 mM NaCl, 5 mM KCl, 1.5 mM CaCl_2_-2H_2_O, 1.2 mM MgSO_4_-7H_2_O, 25 mM NaHCO_3_, 1.2 mM KH_2_PO_4_, 0.03 mM EDTA-Na_2_, 0.6 mM L-ascorbic acid, and 11 mM glucose, pH 7.4). Cells were placed on an inverted fluorescence microscope (Zeiss Axio Observer Z1, Jena, Germany) consisting of an Axio Cam Hsm high-speed monochrome digital camera (Zeiss, Jena, Germany) and a temperature control incubation system to maintain cell viability. To assess the vasoactive response of SMCs in normal and ischemic contexts, a set of experiments were performed. In normal conditions, the non-genomic and genomic effects of RA on vasculature were evaluated. Moreover, the genomic effect of RA on ischemic events was analyzed as follows:

(1) Non-genomic effect of RA on vasculature: The rapid action of 10 µM of RA was analyzed in SMCs pre-contracted with noradrenaline (NA; 1 µM). Controls with the vehicle used to dissolve RA (0.1% ethanol) were always performed. Vascular rapid effects are described as the immediate effects that occur after application of a specific drug on SMCs [25].
(2) Genomic effect of RA on vasculature: For this analysis, SMCs were pre-incubated for 24 h with 0.1% ethanol (control) or with RA at different concentrations of RA (0.1 µM, 1 µM, 5 µM, and 10 µM). Incubation for 24 h is adequate for genomic effects on contractility patterns of SMCs to occur [13]. Then, the vasoactive response of SMCs was assessed using the contractile agent noradrenaline (NA; 1 µM) and the relaxing agent sodium nitroprusside (SNP; 1 µM). Each experiment lasted 60 min. After the first observation, an image of the cells was recorded. For the first 20 min, the cells were kept on the microscope without the addition of any agent. Afterwards, NA was added, and changes in cell area were recorded for 20 min. Then, SNP was added, and changes in cell area were again recorded for 20 min. This period was necessary to obtain a maximum response of the cells to the agents used.
(3) Genomic effect of RA on ischemic events: For this analysis, SMCs were pre-incubated for 24 h with PCC secretome (Table 1). The setup strategy was the same as described in 2).

In all experiments, a reduction in cell area corresponds to a contraction of the cells, while an increase in cell area corresponds to relaxation. Cell area measurement was performed using AxioVision 4.8 software (Zeiss, Jena, Germany).

### 2.5. Drugs

Noradrenaline (NA), sodium nitroprusside (SNP), and RA (reference number R2625) were purchased from Sigma-Aldrich Química (Sintra, Portugal). The stock solutions of NA and SNP were dissolved in distilled water and stored at −20 °C. On the day of each experimental procedure, specific dilutions of these solutions were prepared. RA was diluted in 99.8% absolute ethanol (Sigma-Aldrich, Sintra, Portugal).

### 2.6. Statistical Analysis

Results are expressed as mean ± standard error of the mean (S.E.M.) of at least 3 independent experiments, performed in triplicate. Comparison between more than two groups was analyzed by using a one-way ANOVA followed by Dunnett’s test. Comparisons between the two groups were analyzed with Student’s *t*-test. Differences were considered significant when probability levels were less than 5% (*p* < 0.05). All statistical procedures were performed using the SigmaStat Statistical Analysis System, version 3.5 (2006).

## 3. Results

### 3.1. Evaluation of the Non-Genomic (Rapid) Effects of RA on SMCs

**Figure 1:**
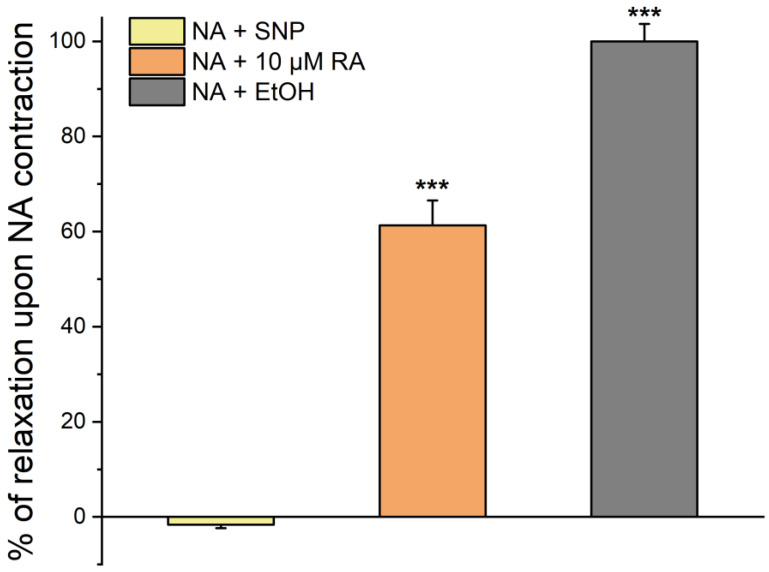
Percentage of SMC relaxation after pre-incubation with NA. Percentage of SMC relaxation induced by ethanol (EtOH), SNP, and RA after a pre-incubation with NA for 20 min. Data are expressed as percentage of cell relaxation relative to the initial area of each cell. Each column represents the mean value ± SEM of three independent experiments performed in quadruplicate. Statistical analysis was performed by one-way ANOVA followed by Dunnett’s test vs. ethanol control, where *** *p* < 0.001.

To evaluate the rapid effect of RA on SMCs, the cells were first stimulated with NA (Figure 1). After 20 min of exposure to NA, the cells were further incubated with 10 µmol/L of RA for 20 min, where a relaxation of 50% was observed. In addition, the addition of SNP promoted a relaxation of 100%.

These results suggest that RA has a clear vasorelaxant effect, although smaller than that observed when using SNP. Controls with ethanol did not modify the vascular tonus.

### 3.2. Evaluation of the Genomic (Long) Effects of RA on SMCs

To analyze the effect of RA on SMC vasoactive response, these cells were pre-incubated for 24 h with different concentrations of RA (0.1 µM, 1 µM, 5 µM, and 10 µM), and its effect on cell area was evaluated using the PCSA technique.

**Figure 2:**
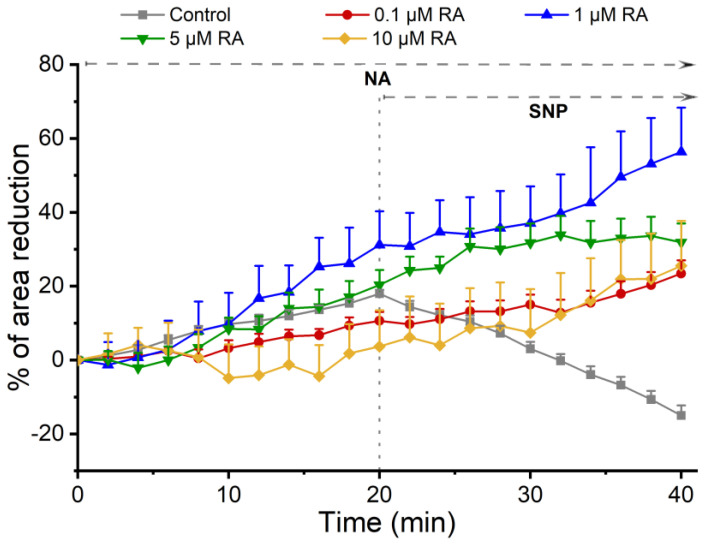
Temporal profile of SMC response to the incubation with RA. Profile of the effect of NA and SNP on SMCs after incubation with different concentrations of RA. Each point represents the mean value, and the vertical lines represent the S.E.M of 3 independent experiments.

Figure 2 shows the temporal profile of the vasoactive response of SMCs when incubated with RA upon the addition of the contractile and relaxing agents. The control group (cells not incubated with RA) showed that NA and SNP promoted SMC contraction and relaxation, respectively. In cells previously incubated with different concentrations of RA, the contractile effect of NA on SMCs was maintained despite this effect being dual and dose-dependent. Indeed, cells pre-incubated with 1 µM and 5 µM of RA increase the NA-induced contraction, and an opposite effect was observed in cells incubated with 0.1 µM and 10 µM, where a decreased contractile stimulus was attained. Concerning the SNP (that is, an NO donor) response, this was unable to induce relaxation, and a contractile response was observed instead.

### 3.3. Effect of RA on the Vasoactive Response Regulation Conditioned by Neurons and Astrocytes

To evaluate the effect of RA on the vasoactive response of SMCs after the occurrence of an ischemic event, SMCs from the MCA were pre-incubated with (reperfusion) media conditioned by co-cultures of neurons and astrocytes previously incubated with different concentrations of RA or the solvent (0.1% ethanol) and submitted to OGD for 4 h.

#### 3.3.1. SMC Vascular Response to NA Contraction

**Figure 3:**
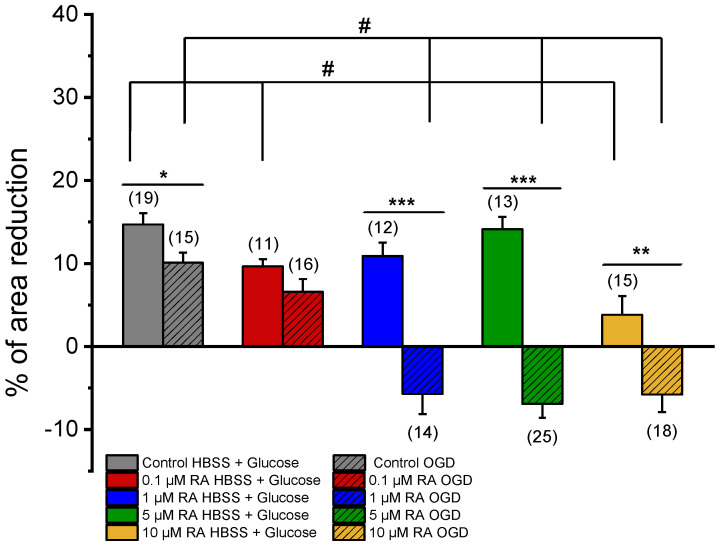
Effect of NA (1 μM) on SMCs. Percentage of NA-induced contraction after 24 h incubation with media conditioned by cortical co-cultures of neurons and astrocytes incubated with RA (0.1 μM, 1 μM, 5 μM, and 10 μM) and subjected (OGD) and not subjected (HBSS + Glucose) to ischemic context and respective controls (co-cultures of neurons and astrocytes not incubated with RA). Data are expressed as percentage (%) change in cell area from the initial area of each cell. Bars represent the mean ± S.E.M of the number *n* within brackets, from three independent experiments (primary cell culture). Statistical analysis was performed by one-way ANOVA followed by Dunnett’s test, where # *p* < 0.05 vs. Control HBSS + Glucose and vs. Control OGD, and by Student’s *t*-test, where * *p* < 0.05; ** *p* < 0.01; *** *p* < 0.001.

Figure 3 represents the effect induced by NA after pre-incubation of SMCs from the MCA with media conditioned by co-cultures of neurons and astrocytes previously treated with RA and subjected (OGD group) or not to an ischemic lesion (HBSS + Glucose group). In addition, the respective controls (ethanol) are also represented (response of SMCs conditioned by the secretome of neuron and astrocyte co-cultures, not incubated with RA, subjected or not to an ischemic lesion). Please see Table 1, where all the solutions used in this study are represented.

NA induced a contraction in SMCs conditioned by OGD (Control = 10.09 ± 1.24% or not HBSS + Glucose Control = 14.70 ± 1.35%), with significant differences between them. Therefore, the secretome of neurons and astrocytes, subjected to ischemic conditions, significantly attenuated the SMC contractile response.

Regarding the response of SMCs not subjected to ischemic lesion, incubation with concentrations of 0.1 μM RA and 10 μM RA resulted in a significant decrease in the SMC contractile response to NA (0.1 μM RA HBSS + Glucose = 9.66 ± 0.86%; 10 μM RA HBSS + Glucose = 3.82 ± 2.27%), when compared to the respective control (Control HBSS + Glucose = 14.70 ± 1.35%) (Figure 3). Thus, as evidenced, upon incubation of these cells with RA, their contractile response decreased.

In OGD conditions, incubations with 1 μM, 5 μM, or 10 μM of RA promoted significant changes in SMC contractile response (1 μM RA OGD = −5.72 ± 2.40%; 5 μM RA OGD = −6.92 ± 1.65%; 10 μM RA OGD = −5.78 ± 2.12%) when compared with the respective control (Control OGD = 10.09 ± 1.24%) (Figure 3). These results evidenced of a transformed response in SMCs when conditioned with RA and subjected to an ischemic lesion. Thus, the use of RA increased the effect of mediators released by neurons and astrocytes subjected to the ischemic lesion, impeding the contractile response of SMCs to NA and leading to relaxation.

#### 3.3.2. SMC Vascular Response to the NO Effect

**Figure 4:**
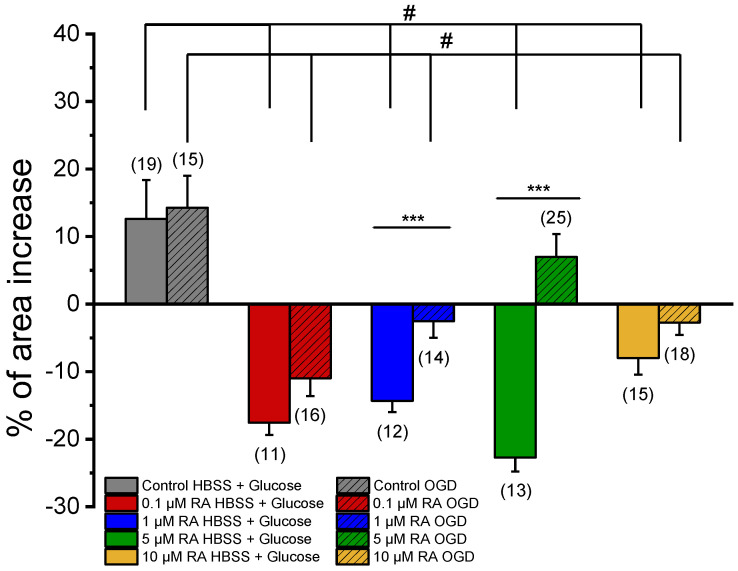
Effect of SNP (1 μM) on SMCs upon contraction with NA. Percentage of SNP-induced relaxation after 24 h incubation with media conditioned by cortical co-cultures of neurons and astrocytes incubated with RA (0.1 μM, 1 μM, 5 μM, and 10 μM), subjected (OGD) and not subjected (HBSS + Glucose) to ischemic context and respective controls (co-cultures of neurons and astrocytes not incubated with retinoic acid). Data are expressed as percentage (%) change in cell area from the initial area of each cell. Bars represent the mean ± S.E.M of the number *n* within brackets, from three independent experiments (primary cell culture). Statistical analysis was performed by one-way ANOVA followed by Dunnett’s test, where # *p* < 0.05 vs. Control HBSS + Glucose and vs. Control OGD, and by Student’s *t*-test, where * *p* < 0.05.

After exposure of SMCs to NA, the effect of SNP was analyzed (Figure 4). This vasorelaxant agent promoted a non-significant increase in the relaxation of the SMCs incubated with media conditioned by PCC cultures subjected to OGD (Control OGD = 14.27 ± 4.74%) when compared with the relaxation of SMCs incubated with media conditioned by co-cultures not subjected to the ischemic lesion (Control HBSS + Glucose = 12.62 ± 5.75%). Please see Table 1, where all the solutions used in this study are represented.

In the cultures not subject to an ischemic environment, incubation with different concentrations of RA caused significant changes in the SMC response to SNP (0.1 μM RA HBSS + Glucose = −17.56 ± 1.81%; 1 μM RA HBSS + Glucose = −14.32 ± 1.70%; 5 μM RA HBSS + Glucose = −22.72 ± 2.07%; 10 μM RA HBSS + Glucose = −8.00 ± 2.46%) when compared to the respective control (Control HBSS + Glucose = 12.62 ± 5.75%). Therefore, as evidenced, in the presence of the vasodilator agent, incubation with different concentrations of RA culminated in SMC contraction; i.e., the effect of RA changes from powerful vasorelaxation to contraction.

The OGD conditions also led to a change in the response of SMCs to SNP. The cultures, previously incubated with 0.1 μM, 1 μM, and 10 μM of RA, resulted in a contractile response of SNP (0.1 μM RA OGD = −10.98 ± 2.64%; 1 μM RA OGD = −2.56 ± 2.42%; 10 μM RA OGD = −2.73 ± 1.83%) when compared to the respective control (Control OGD = 14.27 ± 4.74%). The SMCs incubated with 5 μM RA were the only exception, and the SNP promoted relaxation (5 μM RA OGD = 6.98 ± 3.38%), exhibiting a response similar to the respective control (Control OGD = 14.27 ± 4.74%).

## 4. Discussion

The MCA is the brain artery more frequently affected by ischemia injury [26,27]. Thus, understanding how the adverse environment alters the vasoactive function of MCA-SMCs, as well as developing mechanisms capable of controlling vascular tone, are of utmost importance [25,26].

Thus, this study’s first objective was to evaluate SMC response to RA. It has already been shown that retinoic acid guarantees the integrity of the physiological structure of the neurovascular unit, reducing the development of neurological disorders, and also can modulate the physiological function of the cells of the neurovascular unit, which is crucial for the maintenance of this physiological structure. Moreover, Kang, Park [28] have previously demonstrated that RA reduces the effects of BBB disruption, following the occurrence of an ischemic event, by inhibiting the apoptosis signaling pathway, which is relevant to its influence on stroke. In this sense, the pharmacological use of this compound is under intense investigation, and its effect on SMCs has never been studied [21,28].

The culture method used to obtain SMCs, previously described by Quelhas, Baltazar [25], promotes SMC growth in a synthetic phenotype which does not correspond to the phenotype present in blood vessels—the phenotype responsible for vascular tone control. In this way, 48 h before the experiments, SMCs were exposed to a serum-free medium, for a period of 24 h, in order to promote the phenotype switch to the contractile state [8,25,29].

Through the PCSA technique, the rapid effect of RA on SMCs was first investigated, and vasodilation induced by RA was evidenced. Moreover, to analyze the genomic effects of RA on SMC vasoactive response, these cells were pre-incubated, for 24 h, with different concentrations of RA (0.1 µM, 1 µM, 5 µM, and 10 µM), previously described by Santos, Ferreira [30]. Furthermore, in this study, the concentration spectrum was amplified, and the concentration of 10 µM was used as the highest concentration, since in the article mentioned above, the distribution of RA was performed with nanoparticles, so the distribution was more local, and 10 µM was the limiting concentration with no cytotoxicity effects for cells [30]. The vasoactive response of SMCs was evaluated with the use of NA as a contractile agent and SNP as a relaxing agent. The choice of NA and SNP was based on studies previously performed in this type of cell, where changes in the vasoactive response were demonstrated in the presence of these two agents [13,25]. Briefly, in SMCs, NA acts on adrenergic receptors (α and β), of which α-1A is the most abundant in the brain. The α-1A receptors are associated with Gq proteins, leading to an increase in PLC activity and subsequent formation of DAG and IP3, causing an increase in calcium, resulting in contraction. Regarding β-receptors, these are coupled to Gs protein, which promotes adenyl cyclase activation, leading to an increase in cyclic adenosine 3′,5′-monophosphate (cAMP), which induces vasorelaxation. Regarding SNP, this vasorelaxant agent was used because the NO/sGC/PK/cGMP pathway is the main mechanism associated with the vasorelaxation of these cells and, consequently, with the regulation of blood pressure. As an NO donor, SNP induces activation of sGC, which causes an intracellular increase in Guanosine 3′,5′-cyclic monophosphate (cGMP) production [10,13]. Our data demonstrated that the incubation of the cells with serum-free medium did not compromise the vasoactive response of SMCs, since these cells contracted, in the presence of NA, and dilated, in the presence of SNP, as previously demonstrated by Quelhas, Baltazar [25] and Mariana, Roque [13]. Regarding the incubation of SMCs with the different concentrations of RA, the addition of SNP did not enhance the relaxation of these cells, indicating that incubation with RA altered the vasoactive response of SMCs, possibly through inhibition of NO action, which may impair the blood vessels. Such results suggest that perhaps RA has two effects—the rapid effect, which is non-genomic, and a long-term effect, which is genomic—enhancing SMC relaxation only in the first scenario. The RA treatment led to a loss of the ability of the SNP to induce vessel dilation in the presence of NA-induced contraction. However, the contractile action of NA was not increased for all NA concentrations tested. Indeed, a double dose-dependent effect was obtained, with incubation with RA 1 and 5 µM inducing an increase in contraction with NA, and concentrations of 0.1 and 10 µM inducing a decrease in the contractile stimulus. Typically, when contractions are less evident, the effect of the relaxing agent applied subsequently is always less. This may explain the effects observed for concentrations of 0.1 and 10 µM. However, in cases where RA incubations promoted an increase in NA-induced contractile stimulation followed by SNP-induced contraction, there appears to be a more complex mechanism to explain, and molecular mechanisms should be studied in more detail in future studies.

As previously mentioned, several types of cells can adapt to these conditions during the ischemia period and release several factors that protect the neuronal networks affected by the decreased blood flow [15]. In this sense, to analyze the prophylactic effect of RA on the modulation of the vasoactive response of SMCs by neurons and astrocytes, reperfusion media of co-cultures of neurons and astrocytes, previously incubated with different concentrations of RA and subjected to OGD for 4 h, were used. The period of time that the co-cultures were subjected to an ischemic environment was previously defined by Roque and Baltazar [15], who observed that exposure of these co-cultures to 4 h of OGD did not produce a significant decrease in cell viability when compared to other ischemic times [13].

Our data demonstrated that factors released into the medium, from co-cultures of neurons and astrocytes, not incubated with RA and not subject to ischemic injury, did not compromise the expression or the activity of adrenergic receptors on SMCs, as previously published by Mariana, Roque [13].

Regarding the incubation with RA, the PCC secretome causes a decrease in the NA contractile response, and since the adrenergic receptors mostly involved in the contractile response are the α1-adrenergic receptors, these may be involved in the RA response. However, the action of β-adrenergic receptors in this response cannot be ruled out either.

Afterwards, the contractile response of SMCs was evaluated when exposed to the PCC secretome submitted to the ischemic lesion. Considering that co-cultures were subjected to 4 h of OGD, there would be expected to be an increase in SMC contractile response. However, instead of what was expected, the SMCs’ contractile response was significantly lower compared to their response after exposure to the PCC secretome not subjected to ischemic lesions. Such results contradict previously published data, which demonstrated that the ischemic environment promoted an increase in SMC contractile response [31]. These differences suggest that the factors released by PCC cultures, when subjected to ischemia and followed by reperfusion, appear to alter the adrenergic response of SMCs via α or β-adrenergic receptors. Under similar conditions (OGD group), incubation of the co-cultures with RA seems to promote the release of mediators that condition adrenergic activity. This fact produced not only a suppression of the contractile response but also a slight relaxation of the SMCs.

As previously mentioned, SMCs are the main regulators of blood flow. Therefore, these cells can constrict and relax in order to effectively control vascular tone [10]. In this way, after the evaluation of the contractile response, the ability of SMCs to relax when exposed to the vasodilator agent, SNP, was evaluated. The addition of SNP to SMCs, when previously exposed to reperfusion media conditioned by PCC cultures submitted and not submitted to an ischemic lesion, showed that SMCs remained viable and responsive, since after exhibiting a contractile response to NA, these cells were able to relax upon the addition of SNP. Nevertheless, after exposure of the SMCs to the secretome of PCC previously incubated with RA and not subjected to ischemic lesion, the mediators released by these co-cultures triggered a contractile response in SMCs after the addition of the vasodilator agent. In OGD conditions, their reperfusion medium altered the release of mediators from PCC cultures to the culture medium, conditioning the SMC response to SNP, possibly through the inhibition of NO action. Several studies report that the anti-inflammatory action carried out by RA triggers mechanisms that inhibit NO production either directly [32] or indirectly, through the action of other factors induced by the action of RA, particularly in cultures of astrocytes [16,17]. However, at a concentration of 5 µM RA, SNP-induced relaxation was observed, similar to the control. Indeed, it has recently been shown that in situations of OGD, there is a transient loss of mitochondrial membrane potential, which is induced by transient occlusion. This event permanently damages mitochondria–endoplasmic (ME) reticulum contacts and disrupts Ca^2+^ oscillation in SMCs, the driving force behind spontaneous myogenic vasomotion. In addition, the binding of mitochondria and ER by the specific overexpression of ME-Linker in SMCs restores cytosolic Ca^2+^ homeostasis, remotivates spontaneous myogenic vasomotricity, achieving optimal reperfusion, and improving neurological injury [33]. Therefore, we can hypothesize that this is the mechanism involved in the action of 5 µM AR. On the other hand, at the highest concentration of RA (10 µM), this effect is not observed, possibly due to a maximum effect exerted by RA at a lower concentration, which does not allow the described effect to be realized. Future studies should be carried out to identify the effect of RA on ME-Linker in SMCs and in the restoration of Ca^2+^ homeostasis.

In addition, several studies have shown that RA can both increase and inhibit detoxification pathways following exposure to various xenobiotics or endogenous compounds released during ischemia, including ROS and proinflammatory cytokines. This contradictory effect of RA is apparently due to the activation of RXR or RAR, with RXR activation appearing to benefit the detoxification system and RAR appearing to potentiate harmful effects [22,24]. Furthermore, the selective modulation of RAR and RXR isoforms may explain the contradictory effects observed in different tissues during fundamental physiological or pathological processes, especially during retinoic acid supplementation [22]. The use of selective agonists for RARα isoforms (such as AM 580 and AM 80), RARβ (such as AC 261066 and CD 2314), RARγ (such as AGN204647, CD 437, and BMS 961), and RXRα (such as CD 3254), as well as mixed agonists, such as Adapalene (RARβ and RARγ agonist) and Bexarotene (potent and selective RXR agonist), has been explored in order to modulate these effects. In addition, selective antagonists, such as LY 2955303 (selective RARγ antagonist), HX 531 (potent RXR antagonist), and BMS 493 (pan-RAR inverse antagonist), are important tools for investigating which isoform is involved and how the specific effects of the compound are manifested, particularly when ROS or xenobiotics are present, released during ischemic events, or capable of inducing them [23,34]. The retinoid system plays a crucial role in regulating fundamental physiological processes such as embryonic development and neurodevelopment, and disturbances in this system can lead to unexpected results, such as increased toxicity after ischemia, rather than the expected reduction, highlighting the complexity and challenges in understanding and managing the toxicity associated with retinoid compounds.

## 5. Conclusions

With this investigation, we can conclude that RA produces different effects depending on how it is administered. The rapid effect of RA potentiated the relaxation of SMCs, while the incubation of these cells with RA, for 24 h, promoted SMC contraction when exposed to the action of NA and SNP. Moreover, given that SNP is not only an NO donor (nitrosylating agent) but also releases cyanide, future studies should also include the use of pure NO donors such as MAHMA NONOate, since this undesired release of cyanide together with NO can cause serious adverse effects when used as a drug for the treatment of hypertension or stroke [35,36]. In this sense, the combined use of RA and other pharmacological agents, particularly nitrosylating agents, should be carefully considered and better studied.

Furthermore, the observed results further demonstrate that RA modulated the effect of neuron and astrocyte co-cultures’ secretome on SMCs, altering their response to the vasoactive agents. In summary, the rapid effect of RA appears to have benefits through the relaxation of SMCs, which allows a greater supply of oxygen and nutrients to the brain. Indeed, synthetic forms or analogs of retinoic acid have long been used in the treatment of various diseases, including numerous forms of cancer. RA has also been called a promising target for neuroprotective therapy of intracerebral hemorrhage [37]. For this reason, RA needs to be more widely studied as a neuroprotective drug, including in the prevention of ischemic stroke. However, continued supplementation of RA shows negative effects on SMC response after the occurrence of an ischemic stroke, similar to what happens in other parts and pathologies of the human body [37]. Therefore, the use of this agent as a drug for the treatment of stroke should only be considered as a treatment immediately after the event, and its prolonged use may be a good method for neuroprotection, namely the constriction of SMCs, which may be beneficial for the prevention of intracerebral hemorrhage events. However, future studies on RA are urgently needed for safe therapeutic use.

## Figures and Tables

**Table 1:** Different media used in the SMC incubations. Legend: Reperfusion medium conditioned (RMC) and oxygen and glucose deprivation (OGD).

Condition	RA Incubation	Medium
HBSS + Glucose	0.1% ethanol (solvent control)	No-OGD-treated RMC
	0.1 µM RA	No-OGD-treated RMC
	1 µM RA	No-OGD-treated RMC
	5 µM RA	No-OGD-treated RMC
	10 µM RA	No-OGD-treated RMC
OGD (4 h)	0.1% ethanol (solvent control)	OGD-treated RMC
	0.1 µM RA	OGD-treated RMC
	1 µM RA	OGD-treated RMC
	5 µM RA	OGD-treated RMC
	10 µM RA	OGD-treated RMC

## Data Availability

The original contributions presented in this study are included in the article. Further inquiries can be directed to the corresponding authors.

## Abbreviations

SMC	Smooth muscle cells
RA	Retinoic acid
NVU	Neurovascular unit
EC	Endothelial cells
BBB	Blood–brain barrier
CBF	Cerebral blood flow
CNS	Central nervous system
NO	Nitric oxide
RAR	Retinoic acid receptor
RXR	Retinoic X receptor
MCA	Middle cerebral artery
PBS	Phosphate-buffered saline
CCM	Complete culture medium
FBS	Fetal bovine serum
FGF	Fibroblast growth factor
NBM	Neurobasal medium
OGD	Oxygen and glucose deprivation
HBSS	Hank’s buffered saline solution
PCSA	Planar cell surface area
NA	Noradrenaline
SNP	Sodium nitroprusside
sGC	Soluble guanylate cyclase
